# A super SDM (species distribution model) ‘in the cloud’ for better habitat-association inference with a ‘big data’ application of the Great Gray Owl for Alaska

**DOI:** 10.1038/s41598-024-57588-9

**Published:** 2024-03-27

**Authors:** Falk Huettmann, Phillip Andrews, Moriz Steiner, Arghya Kusum Das, Jacques Philip, Chunrong Mi, Nathaniel Bryans, Bryan Barker

**Affiliations:** 1 -EWHALE Lab-, Biology and Wildlife Department, Institute of Arctic Biology, University of Alaska, Fairbanks, AK 99775 USA; 2 Department of Computer Science and Engineering, University of Alaska, Fairbanks, AK 99775 USA; 3 Indigenous Health, Institute of Arctic Biology, University of Alaska, Fairbanks, AK 99775 USA; 4 National Academy of Sciences, Beijing, China; 5 Oracle for Research, 2300 Oracle Wy, Austin, TX 78741 USA

**Keywords:** Big data, Machine learning ensemble, Open access, Open source geographic information system (OGIS, QGIS), Great Gray Owl (*Strix nebulosa)*, Alaska, Cloud computing, Oracle cloud infrastructure, Conservation biology, Ecological modelling, Environmental sciences

## Abstract

The currently available distribution and range maps for the Great Grey Owl (GGOW; *Strix nebulosa*) are ambiguous, contradictory, imprecise, outdated, often hand-drawn and thus not quantified, not based on data or scientific. In this study, we present a proof of concept with a biological application for technical and biological workflow progress on latest global open access ‘Big Data’ sharing, Open-source methods of R and geographic information systems (OGIS and QGIS) assessed with six recent multi-evidence citizen-science sightings of the GGOW. This proposed workflow can be applied for quantified inference for any species-habitat model such as typically applied with species distribution models (SDMs). Using Random Forest—an ensemble-type model of Machine Learning following Leo Breiman’s approach of inference from predictions—we present a Super SDM for GGOWs in Alaska running on Oracle Cloud Infrastructure (OCI). These Super SDMs were based on best publicly available data (410 occurrences + 1% new assessment sightings) and over 100 environmental GIS habitat predictors (‘Big Data’). The compiled global open access data and the associated workflow overcome for the first time the limitations of traditionally used PC and laptops. It breaks new ground and has real-world implications for conservation and land management for GGOW, for Alaska, and for other species worldwide as a ‘new’ baseline. As this research field remains dynamic, Super SDMs can have limits, are not the ultimate and final statement on species-habitat associations yet, but they summarize all publicly available data and information on a topic in a quantified and testable fashion allowing fine-tuning and improvements as needed. At minimum, they allow for low-cost rapid assessment and a great leap forward to be more ecological and inclusive of all information at-hand. Using GGOWs, here we aim to correct the perception of this species towards a more inclusive, holistic, and scientifically correct assessment of this urban-adapted owl in the Anthropocene, rather than a mysterious wilderness-inhabiting species (aka ‘*Phantom of the North*’). Such a Super SDM was never created for any bird species before and opens new perspectives for impact assessment policy and global sustainability.

## Introduction

Knowing where animals occur is a crucial component in our understanding of a science-based conservation management and global sustainability in the real industrial world; the Anthropocene and its challenges (e.g.^1,2^). Methods to obtain such knowledge are commonly not robust nor very advanced. As per textbook (see for instance^3^), they are primarily based on inappropriate linear functions^4^^.^, simplistic use of step-wise coefficients^5^, frequency statistics and parsimony, unrealistic parametric assumptions, simplistic computing, and the use of relatively few predictors widely 'underdescribing' and biasing ecology (e.g. < 5 predictor variables); examples shown in^6,7, 8^. These problems are well-known and described for decades (e.g.^4,9–12^), not reflecting well on a modern science-based management employing readily-available computer models and what complex ecology with a myriad of linkages, or reality, really is about. Required progress has been widely insufficient^1,2, 12^. A good example for dealing better with ecological complexities is already telecoupling and spill-over effects^13^. But while widespread and freely available for already over two decades, more holistic methods like machine learning algorithms^14,15^, ensemble models^16-18^ and supercomputing based on widely available open access ‘Big Data’ are still widely ignored^19-21^, underused and not applied to their potential (^11^ and citations within), e.g., multivariate analysis done with modern methods (^22^; see^23^ for a national application in the subarctic). Considering the global environmental crisis^12^, so far, the progress in such globally relevant fields like conservation policy based on multivariate efforts have been quite insignificant (e.g.^1,2, 11^). For instance, most species management models still remain in the single-species realm ignoring species clusters and communities (^11^, see^7^ for Resource Selection Funcstions RSF, and^4^ for Habitat Suitability Index HSI). Also, telemetry data and geolocator data for most of the species are still missing and widely biased for sample sizes and animal strata, frequently  still hand-mined for perceived outliers or using ‘an assumed common-sense’ code (example shown here^24^ and with an application by^25^). It is clear that the sheer magnitude and complexity of biodiversity cannot be geo-tagged for a solution, nor should. Promoting more geo-tagging efforts and mindsets for a proper science, and conservation remains far away from the realistic and natural species distribution and from global realities. Lacking already a relevant consideration of scale and autocorrelation those approaches  do not achieve any modern modeling concepts for urgently needed population-inference in times of the global biodiversity crisis. It just remains in a repetitive ‘me too’ point-and-click science ‘group-think’. Such a low-performing institutional culture - without deeper reflection on progress—a missing vision—still dominates, e.g., in regular SDMs the use of just a few predictors and Maximum Entropy (Maxent) (= a shallow learning machine learning algorithm,^26,27^). A relevant research design with relevant strata, a mutually accepted taxonomy for sampling, meaningful absence and availability data linked with socio-economic or higher precision climate change predictors all rule in their absence. For mandated biodiversity management this is often widely impossible to achieve even. The codified species-habitat models like HSIs, RSFs, Occupancy Models^28^ or Species Distribution Models (SDMs;^29^) are widely competing with each other, are often not in mutual agreement and still use methods being at least 20 years old (^11^^, and citations within^), e.g., Maxent as a leading algorithm in regular SDMs (^26,27, 29^; Maxent as an algorithm comes from the 1960s and was not improved in relevant terms since the 1980s still remaining in the probability framework based on parametric assumptions, which are dubious to obtain in real-life biology, e.g.^4,11^). Instead, modern ensemble model approaches that are based on J. Friedman’s paradigm of ‘*many weak learners make for a strong learner*’ are far and few but powerful (^30; see also 11^). For HSIs, RSFs and Occupancy Models—still widely taught and used in the wildlife discipline, its institutions and federal contractors applied for governance policy—the reality is even worse (based on ambiguous parsimony, linearity, few predictors and dubious model fittings for probability requiring a strict but unrealistic and rarely achieved research design;^4, 11, 28^ respectively).

In the meantime, with open access data sources on the rise in the Anthropocene, many managed species are now of great concern and the wider ecology is simply left unaddressed, still using an underlying governance understanding and policy that comes from over 100 years ago (see here the dominant legal interpretation of ‘*Originalism*’^31^, see^32^ for a critique and failure). It does not remotely allow for modern, latest, or more relevant telecoupling approaches^13^ and similar (see^33^ for Deep Ecology and holistic aspects) in the world we actual live in (‘*the Anthropocene*’), or for massive problems faced by humanity in the future.

Employing best-available methods for confidence of the inference^11^, being accurate and precise matters for a proper habitat and species management^3^. That concept applies even more so in areas that are already deeply affected by the Anthropocene^20,21^, as well as with a human-accelerated climate change where a vast environmental onslaught is predicted to occur. Sophistication matters for a good outcome.

Using a new and best-available large open access global geographic information system (GIS) predictor data set for Alaska, here we introduce and show an example of improved options available: Super SDMs (^34^, for regular and latest SDMs see^35–37^, as well as^23, 27^. Here we apply it for a species paradox, the charismatic and circumpolar but greatly unknown, understudied and misunderstood so-called ‘*Phantom of the North*’ (https://abcbirds.org/bird/great-gray-owl/;^38^)—the Great Gray Owl (*Strix nebulosa*). It is a very popular species in the public eye (see for instance featured in '*Into the Wild*' movie and book for remote Alaska^39^). This species is likely long-lived and has a circumpolar distribution^38^. Relevant distribution data for this species are scarce and widely missing though in Alaska^40,41^. We introduce here the generic concept of a ‘Super SDM’^34^ based on a widely extended set of open access predictors and latest computational methods. We investigate and promote it as a new but readily available science-mandated global baseline for inference in species-habitat associations. Knowing best-available species-habitat associations are of crucial importance on a finite planet, while consumption patterns, human population, social inequality, habitat fragmentation, sea levels, global temperatures, etc. are greatly on the rise compromising wilderness and its species.

## Methods

**Figure 1:**
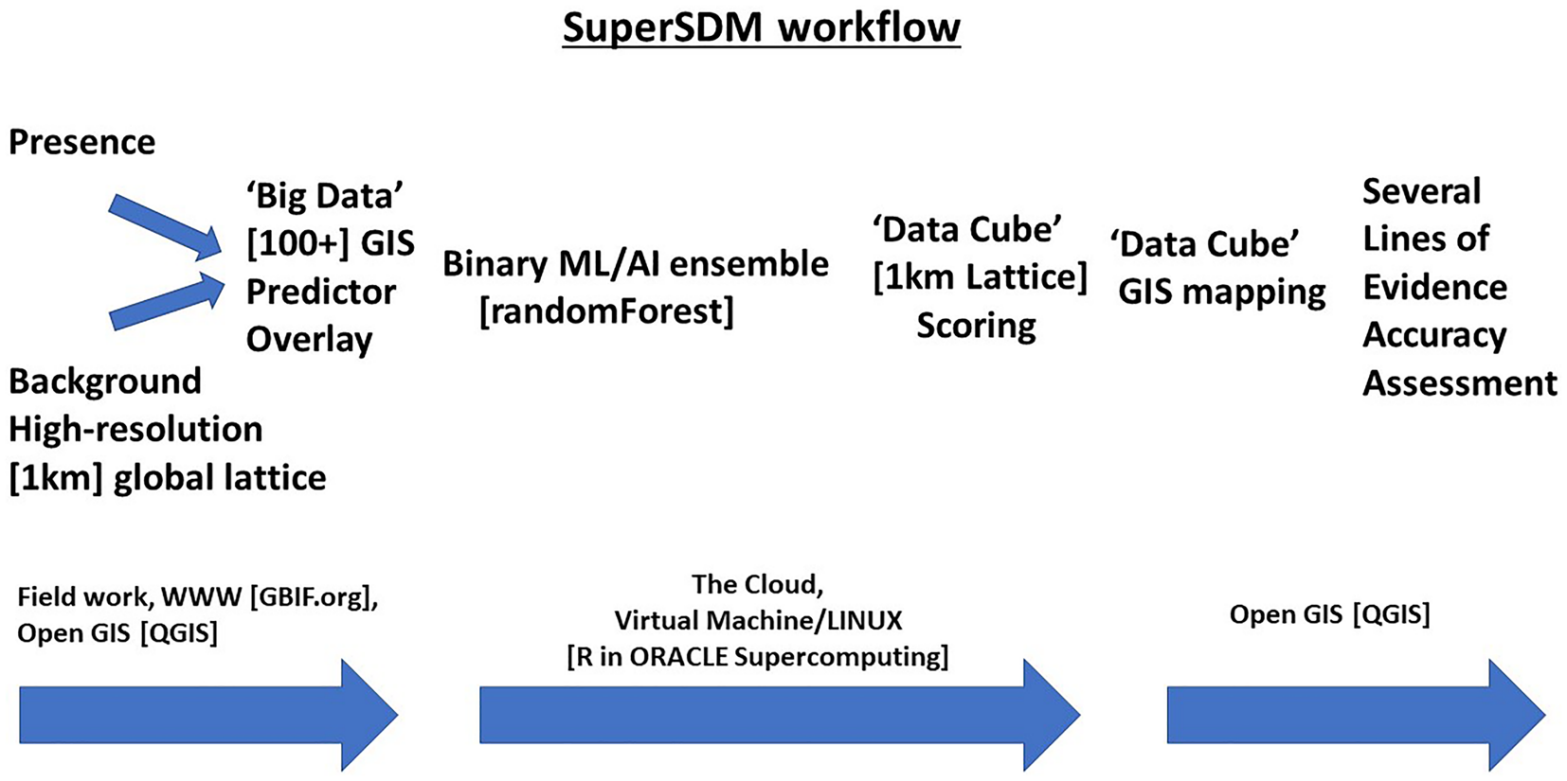
Generic workflow for this study and suggested for SuperSDMs. Text in brackets has adjustable components and as were used in this study).

We started with the pioneering study approach presented by^42^, based on^34, 35^) and applied it as an update to Great Gray Owls (GGOW; taxonomic serial number TSN 177929) for Alaska. It followed the initial work from^43^ and then got extended with more and fine-tuned predictors and a cloud computing platform to overcome computing limitations towards progress. The workflow is described below and visualized in Fig. 1.

### Data

**Figure 2:**
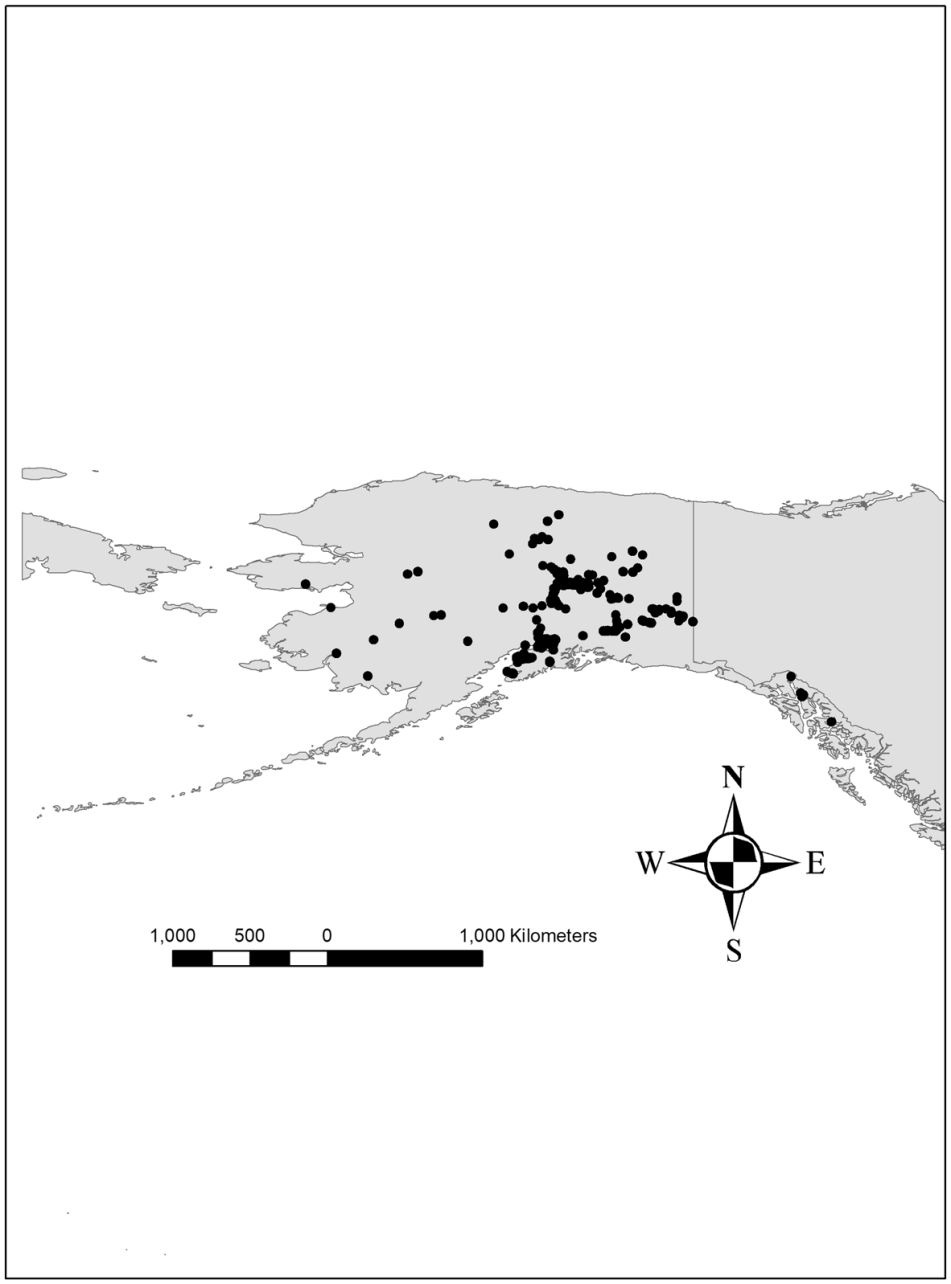
Great Gray Owl sightings in the study area of Alaska.

We compiled likely the best-known and publicly available open access occurrence records for GGOWs in Alaska (n = 410), covering years from 1880 til 2019 (see Fig. 2); virtually all data points come from visual detections; whereas relevant nest location information are widely unknown in Alaska and unlikely for those data. The data are in the public domain (see^43,44^ for citizen science data), got merged from various publicly-available sources and do not carry a unifying underlying protocol and research design (details in^43^; eBIRD citation provided further below). Because we let the algorithm take care of data and outliers for generalization (sensu^11^), we do not filter the precious data. Still, wrong identifications and erroneous species confusions for GGOW are virtually impossible due to its unique appearance (for more data validity details see^43,44,45^). GGOWs are not known to occur in clusters and usually found individually^46^, thus autocorrelation is not an apparent issue for this species and its data (our model analysis of ‘tree-based algorithms’ is relatively robust to such issues regardless, see^11^), and citations within. These presence data were merged with the ‘background data’ (pseudo-absence) for all of the study area resulting in a binary response (presence/absence) for the subsequent data mining and models based on a relative index of occurrence (RIO;^11^).

In addition, we also compiled the best-available global open access set of GIS layer predictors. Here we used Alaska as the study area, environmentally described by 100+ predictors (‘Big Data’; we currently have an even larger global data set of over 132 and of 230 GIS layers^33^), but here we focus on Alaska-specific questions and use its continuous predictors (while many other categorical predictors remain unused, still awaiting their use and further assessment). The list of utilized predictors can be seen in Table 1. This dataset exists in the form of ASCII/TIFF files in a WGS 1984 geographic projection of latitude and longitude in decimal degrees (see Data Availability section and Appendix section within). For layer creation of the specific Alaska features we used also the Alaska state NAD1983 projection with coordinates in feet for a slightly higher accuracy of local variables.

**Table 1:** List of predictors for Alaska used in this study; the majority of predictors are climate-related (6 datasets with monthly mean metrics; n = 75) with some topographic (n = 5), biological (n = 5) and human-related ones (n = 15). This data set is a dynamic Open Access GIS layer dataset compiled by Sririam and Huettmann (unpublished, Andrews 2019 and Steiner and Huettmann in review). It lists overall more than 219 GIS Layers for Alaska.

Data set #	Data	Res	Units	Variable type	Specific source	Citations
1–12	Average Temperature by month^12^	60 m	C*100	Quan	PRISM	Sriram and Huettmann (unpublished)
13–24	Average precipitation by month^12^	60 m	Mm	Quan	PRISM	Sriram and Huettmann (unpublished)
25	Human population density	1 km	Humans/km^y^	Quan	ICESIN	Sriram and Huettmann (unpublished)
26	NDVI	1 km	Index	Quan	Website	Sriram and Huettmann (unpublished)
27	Globcover	1 km	Categories	Cate-gorical	Website	Sriram and Huettmann (unpublished)
28	GLC2000	1 km	Categories	Cate-gorical	Website	Sriram and Huettmann (unpublished)
29–41	Cloudcover by month	60 m	%	Quant	World Clouds	Sriram and Huettmann (unpublished)
42–61	BIOCLIM 1–19	1 km	Indeces	Quan	Bioclim	Sriram and Huettmann (unpublished)
62	Aspect	300 m	Degrees	Quan	USGS	Sriram and Huettmann (unpublished)
63–75	Solar radiation by month	1 km	Kjul	Quan	World Solar Radiation	Sriram and Huettmann (unpublished)
76	Human Footprint	2 km	Index	Rank	Assembled	WWF
77	Mammal density	2 km	Species number	Quan	Publication	Steiner and Huettmann (in review)
78	Bird density	2 km	Species number	Quan	Publication	Steiner and Huettmann (in review)
79	Proximity to coast	1 km	Index (km)	Quan	GIS	Andrews (2019)
80	Lake proximity	1 km	Index (km)	Quan	GIS	Andrews (2019)
81	Road proximity	1 km	Index (km)	Quan	GIS	Andrews (2019)
82	Proximity to ‘water’	1 km	Index (km)	Quan	GIS	Andrews (2019)
83	Proximity to Airport	1 km	Index (km)	Quan	GIS	Andrews (2019)
84	Proximity to Fire	1 km	Index (km)	Quan	GIS	Andrews (2019)
85	Proximity to pipeline	1 km	Index (km)	Quan	GIS	Andrews (2019)
86–98	Monthly global mean temperatures	1 km	Deg C	Quan	World Climate	Sriram and Huettmann (unpublished)
99	World Rodent Diversity	2 km	Species number	Quan	Publication	Steiner and Huettmann (in review)
100	Elevation	300 m	M asl	Quan	USGS	Steiner and Huettmann (in review)
101	Model1	1 km	RIO	Quan	Publication	Zahibi et al. (2091(
101	X coordinate	M		Quan	GIS	Not used in models as a predictor
102	Y coordinate	M		Quan	GIS	Not used in models as a predictor

We then used a point lattice of 1 km for Alaska, created in Open GIS  QGIS (vers. 3.28 Firenze; https://blog.qgis.org/2022/10/25/qgis-3-28-firenze-is-released/). Those lattice points were used as background (pseudo-absence) samples to be compared with presence points in the study area as part of a binary response (see also^11,47^). But also it was later used as a point-prediction grid for the study area for overlays with the predictors (resulting in the ‘data cube’). That way it was also used for scoring the predictions from the model described below to each lattice point (as presented in^11^). This step is crucial to geo-reference the obtained predictions, allowing for a spatial representation of the model results. The data cube is exported as a stand-alone table in a CSV format consisting of 373,423 rows (lattice points) and 105 columns and has a size of 206 MB.

Thanks to the machine learning approach used here, one is able to handle all the compiled data, including some potentially uncertain data (aka ‘bad apples’; see^11^ and citations within). Thus, we did not engage much into specific data cleaning, transformation or correction of the raw data (= GGOW locations and predictors). Being able to use default data speaks to the powerful research design we allow, and here we relied on data sections received (e.g. openly shared with the global public) and brought together. In this study we actually let the algorithm ‘learn’ the signals in the data and handle all the data realities for generalization (sensu^48,49^; “*inference from predictions*” as a core scheme of the approach chosen and promoted by Leo Breiman; see also^11^ and citations within). We then assess the major predictions with a test using several lines of evidence to convince. Here we apply published and alternative data, e.g. coming from a research design, as well as several citizen science source data for this species overall within Alaska (examples show in^50^).

### Models and cloud computing

For a proof of concept, we used a basic RandomForest (‘bagging’, a powerful ensemble model classifier;^48-51^) run in R on the data cube. In order to successfully run this analysis, we utilized the R packages ‘randomForest’ (https://cran.r-project.org/web/packages/ randomForest/ index.html; see^52,53^ for further justification of this application). We followed Formula 1 for a RandomForest run. Details of the base code we used in R are shown in Appendix 1 (see Data Availability section).

$$\begin{aligned} {\text{Formula 1}}:\quad & {\text{Presence}}/{\text{Background }}\sim {\text{ tmean}}\_{\text{1 }} + {\text{ tmean}}\_{\text{2 }} + {\text{ tmean}}\_{\text{3}} + {\text{tmean}}\_{\text{4}} + {\text{tmean}}\_{\text{5}} \\ & + {\text{tmean}}\_{\text{6}} + {\text{tmean}}\_{\text{7}} + {\text{tmean}}\_{\text{8}} + {\text{tmean}}\_{\text{9}} + {\text{tmean}}\_{\text{1}}0 + {\text{tmean}}\_{\text{11}} + {\text{tmean}}\_{\text{12}} \\ & + {\text{prec}}\_{\text{1}} + {\text{prec}}\_{\text{2}} + {\text{prec}}\_{\text{3}} + {\text{prec}}\_{\text{4}} + {\text{prec}}\_{\text{5}} + {\text{prec}}\_{\text{6}} + {\text{prec}}\_{\text{7}} + {\text{prec}}\_{\text{8}} + {\text{prec}}\_{\text{9}} \\ & + {\text{prec}}\_{\text{1}}0 + {\text{prec}}\_{\text{11}} + {\text{prec}}\_{\text{12}} + {\text{pdensit1}} + {\text{ndvi}} + {\text{globcover}} + {\text{ glc2}}000 + {\text{cloud1}} \\ & + {\text{cloud2}} + {\text{cloud3}} + {\text{cloud4}} + {\text{cloud5}} + {\text{cloud6}} + {\text{cloud7}} + {\text{cloud8}} + {\text{cloud9}} \\ & + {\text{cloud1}}0 + {\text{cloud11}} + {\text{bio}}\_{\text{1}} + {\text{bio}}\_{\text{2}} + {\text{bio}}\_{\text{3}} + {\text{bio}}\_{\text{4}} + {\text{bio}}\_{\text{5}} + {\text{bio}}\_{\text{6}} + {\text{ bio}}\_{\text{7}} \\ & + {\text{bio}}\_{\text{8}} + {\text{bio}}\_{\text{9}} + {\text{bio}}\_{\text{1}}0 + {\text{bio}}\_{\text{11}} + {\text{bio}}\_{\text{12}} + {\text{bio}}\_{\text{13}} + {\text{bio}}\_{\text{14}} + {\text{bio}}\_{\text{15}} \\ & + {\text{bio}}\_{\text{16}} + {\text{bio}}\_{\text{17}} + {\text{bio}}\_{\text{18}} + {\text{bio}}\_{\text{19}} + {\text{ aspect}} + {\text{solrad1}} + {\text{solrad2}} + {\text{solrad3}} \\ & + {\text{solrad4}} + {\text{solrad5}} + {\text{solrad6}} + {\text{solrad7}} + {\text{solrad8}} + {\text{solrad9}} + {\text{solrad1}}0 + {\text{ solrad11}} \\ & + {\text{solrad12}} + {\text{hf}} + {\text{mammals}} + {\text{birds}} + {\text{distcoasta}} + {\text{distlakeri}} + {\text{ EucDistTow}} \\ & + {\text{EucDstAirp}} + {\text{EucDistFir}} + {\text{DistPipeli }} + {\text{ World}}\_{\text{MIN1}} + {\text{ World}}\_{\text{MIN2}} \\ & + {\text{World}}\_{\text{MIn3}} + {\text{World}}\_{\text{MIn4}} + {\text{ World}}\_{\text{MIn5}} + {\text{World}}\_{\text{MIN6}} \\ & + {\text{World}}\_{\text{MIN7}} + {\text{World}}\_{\text{Min8}} + {\text{World}}\_{\text{Min9}} + {\text{World}}\_{\text{Min1}}0 \\ & + {\text{World}}\_{\text{Min11}} + {\text{World}}\_{\text{Min12}} + {\text{GlobalRive}} + {\text{WorldSlope}} \\ & + {\text{WorldRoden}} + {\text{WorldSoil2}} + {\text{Model1}} \\ \end{aligned}$$

Using these data initially on a consumer-grade laptop (16 GB memory), we ran into a run-time memory error indicating that it is not executable on a common laptop machine, and thus, cannot be completed as a model prediction without removing data or simplifying the prediction model. This is a bottleneck, thus far, not allowing to progress. So here we tried to overcome this computing bottleneck with super computing in a cloud-computing environment from the Oracle Cloud Infrastructure (an Oracle for Research computing credit grant provided to FH).

An Oracle Cloud virtual machine instance running Oracle Linux 8 was accessed via SSH through Windows Powershell. Installed on the machine was R 4.2.2. Details of the virtual machine are shown in Table 2. Those settings are not on the extreme side of cloud-computing but are sufficient to have the RandomForest run completed on the Big Data set that otherwise would not have been solved. It presents a showcase of the feasibility, magnitude, and potential of the workflow presented in this study, allowing many subsequent applications and presenting vast potential.

**Table 2:** Supercomputing settings.

Oracle cloud metric	Description
Computer system	Linux
Memory (CPU Capacity)	1024 GB
OCPU count	64
Machine shape	VM.Standard.E4.Flex
Internet bandwidth	40 Gbps
Cores	AMD EPYC 7113

### Model assessment

For a robust inference, model predictions are to be assessed for validity^11^. Ideally, that’s done with different lines of evidence. While we have exhausted all known publically-available data sources for this species, as available in GBIF.org and^43^, here we inquired with several alternative and more recent data sources beyond 2019, such as vetted bird watching listervs and citizen science web portals, e.g. iNaturalist (https://www.inaturalist.org/; new data collected).

## Results

### Data

We were able to compile the best publicly available distribution occurrence dataset for Great Gray Owls (GGOW) in Alaska; it covers a unique time period from 1880 to 2019, and is a testable quantified research component useable as a point data set (n = 410) in a CSV (ASCII) format, originating from various sources now existing as a GIS shapefile (see in Data Availability section, Appendix 3a within).

Further, we compiled, and make, the entire underlying GIS predictor set of over 100 GIS layers for Alaska available (see in Data Availability section, Appendix 2 within).

Both data sets are described with FGDC ISO compliant metadata in XML & HTML format (see also as part of the respective Data Availability section, Appendix within) to understand the data making it an inherent outcome of this multi-year study.

### Model run

**Figure 3:**
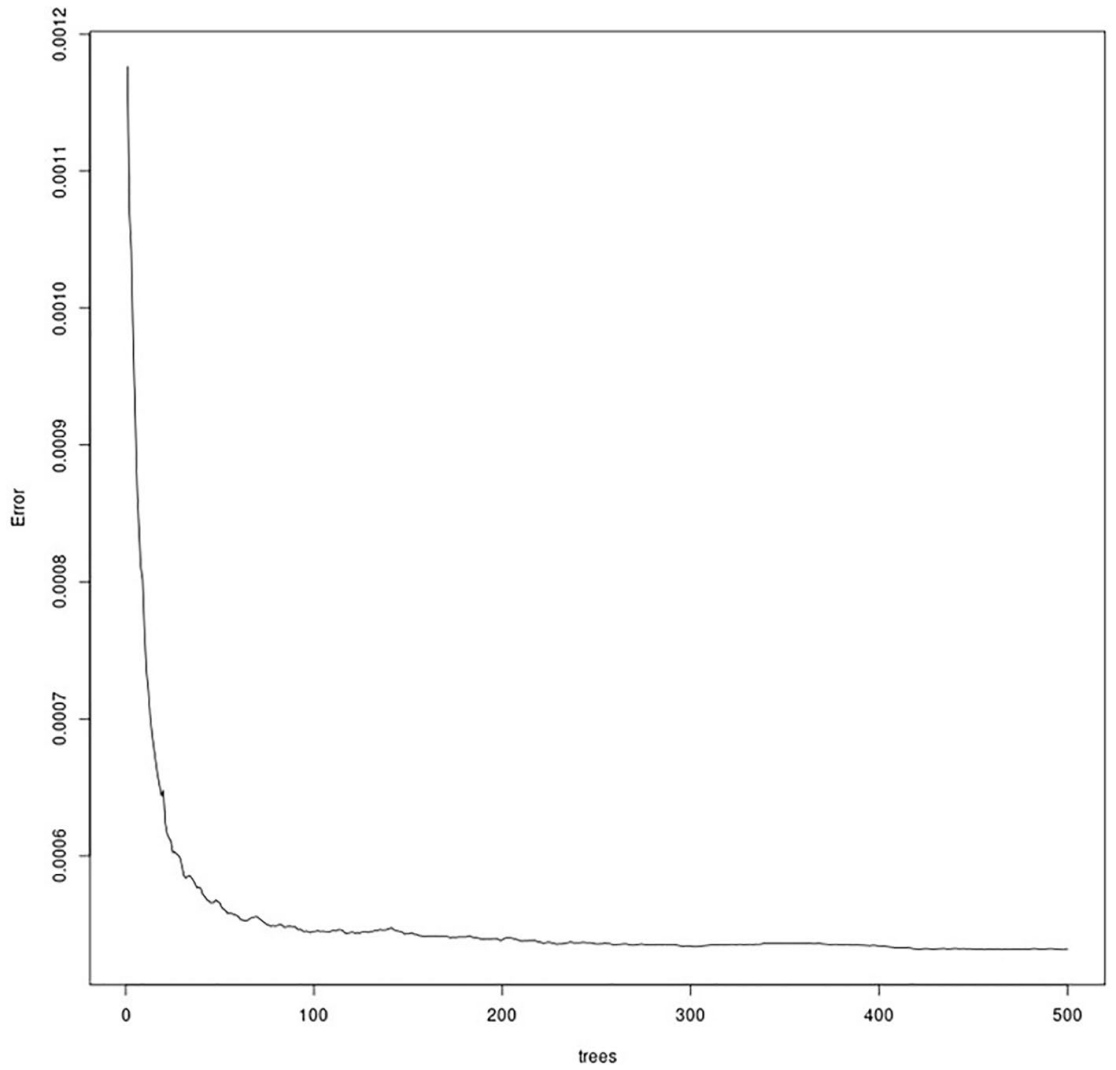
Randomforest Model fit (error) by number of trees showing a good and fast model fit.

For the first time, we were able to complete an open access and open source workflow using Big Data for GGOW for a basic ensemble model algorithm (RandomForest) in the R environment run on a cloud computing workstation. We got a good model conversion (Fig. 3). This model ran c. 8 h, some of the figures required another overall 1 h to complete. The memory usage of the model run is up to 80% (of the assigned 1,024 GB).

**Figure 4:**
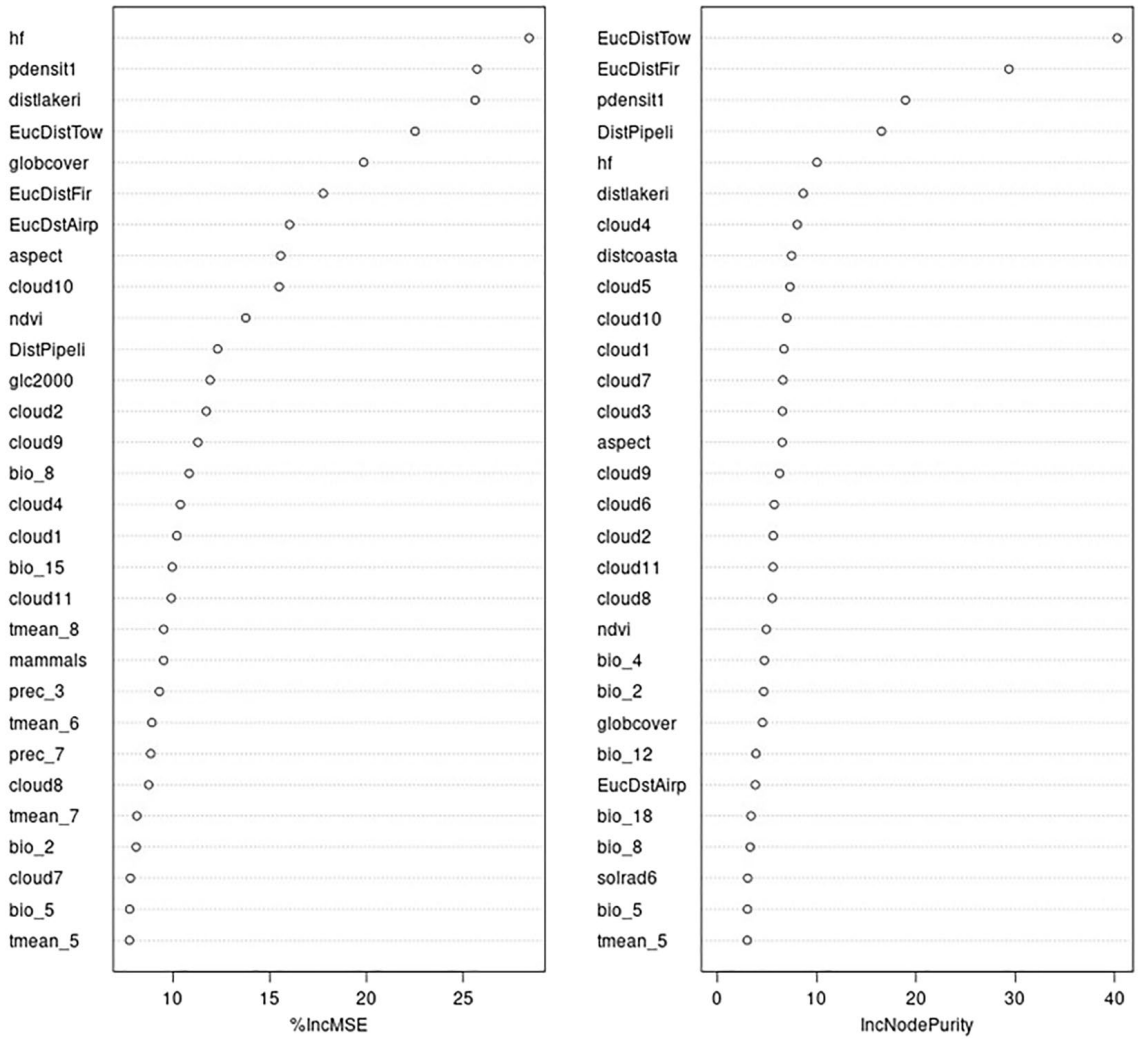
Variable importance using two metrics (MSE, node purity) showing a variety of ecological predictors driving the GGOW occurrence with some predictor groups dominating, e.g. human impacts.

**Figure 5:**
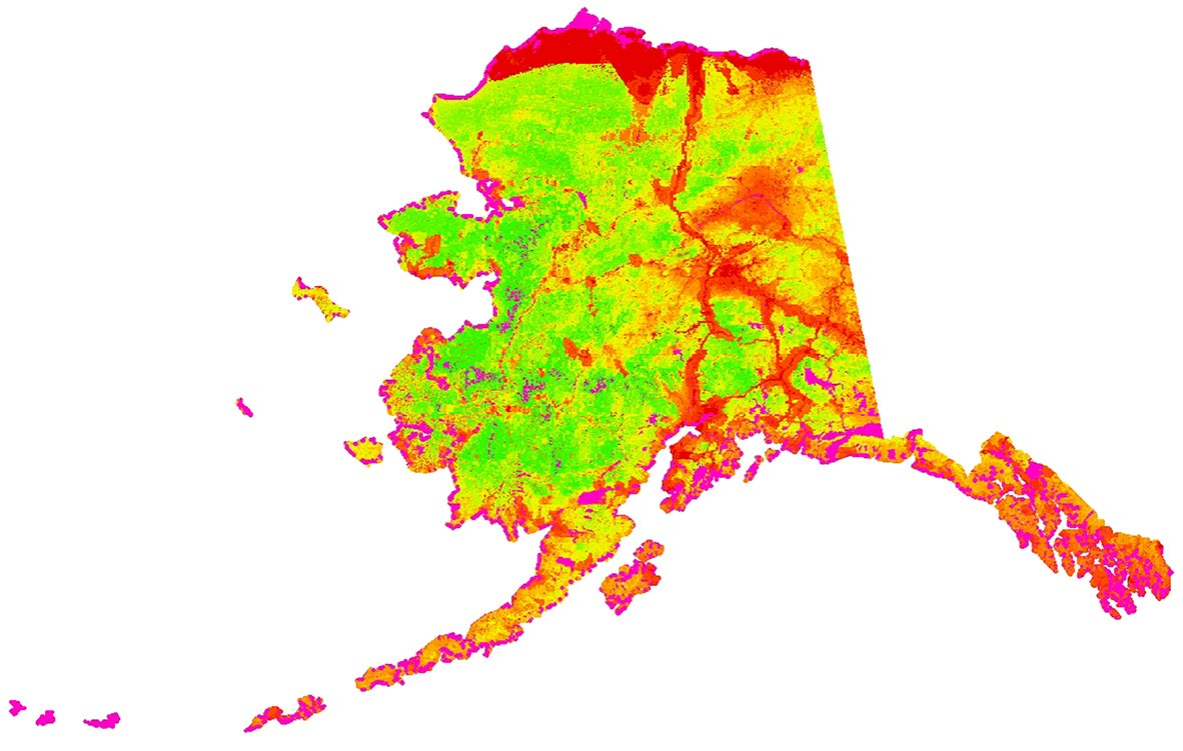
Great Gray Owl raw predictions in the study area of Alaska using randomForest; the relative index of occurrence (RIO) is shown along a color gradient of red (predicted presence) and green (predicted absence).

Figure 4 shows the variable importance ranks of the 100 predictors we used, which presents the basis for the subsequent predictions (Fig. 5) and are further discussed in the next section for their meaning.

### Model predictions and accuracy

The map shown in Fig. 5 is the first prediction using machine learning ensembles and Big Data ever completed for Great Gray Owls (GGOWs) in Alaska and around the globe using a cloud-computing environment.

Our prediction result shows hotspots and coldspots for GGOWs in Alaska; the state with the largest protected area system in the U.S. However, our predicted ecological niche of GGOW does not match well with traditional range maps: in the predicted ecological niche the hotspots are primarily found along roads and urban areas, as well as human settlements (villages) and industrial areas, including some coastal zones and the Arctic tundra. Whereas the predicted coldspots are seen in western Alaska and in other vast sections of Alaska’s wilderness, including many protected areas and some wilderness regions. According to the predicted ecological niche (as per^11^ and citations within) transferred from the geographic niche this is a robust quantifiable finding to test further (details shown below for evidence and confidence).

**Figure 6:**
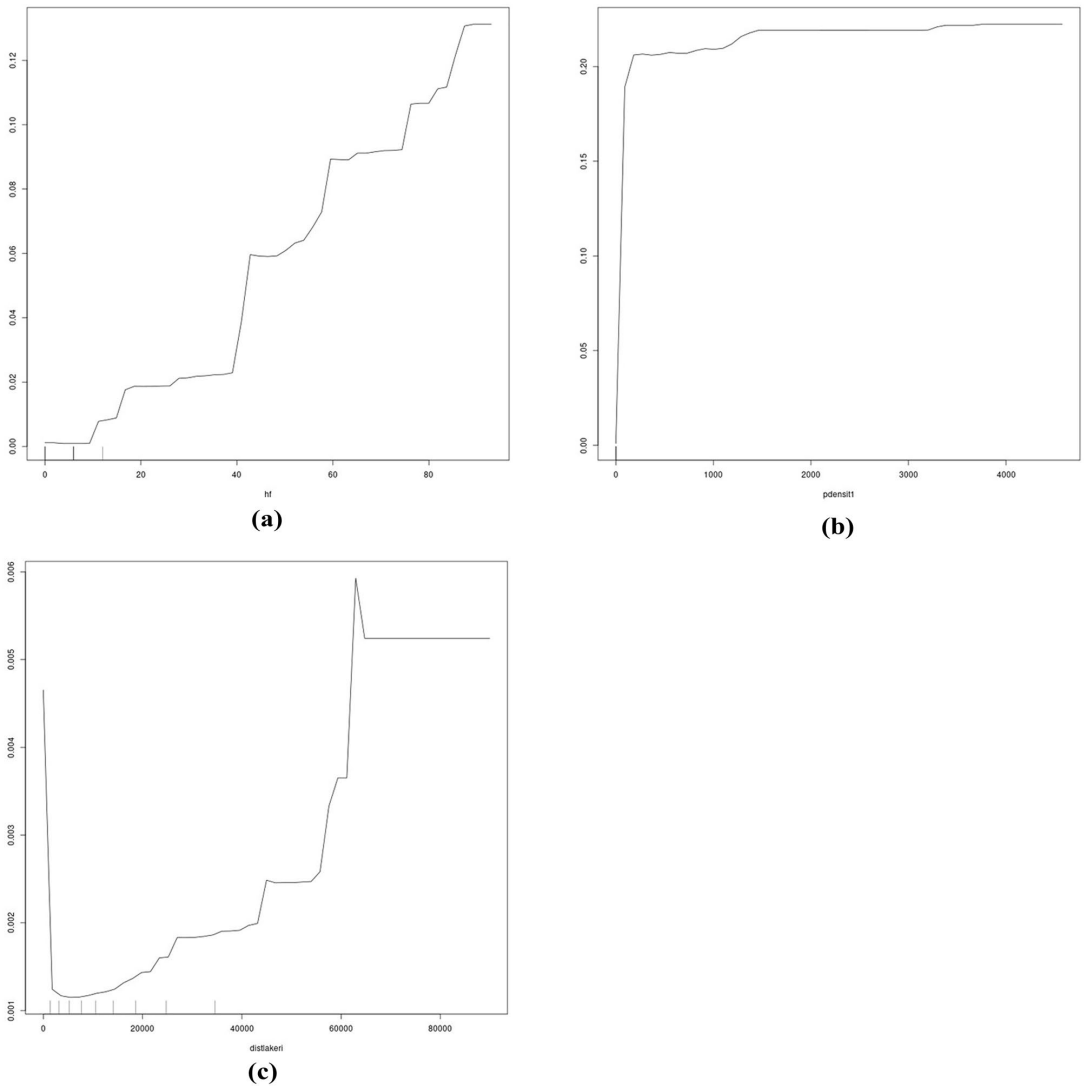
(**a**–**c**) Partial dependence plot of the topthree predictors using MSE (hf, pdens, hlake).

**Figure 7:**
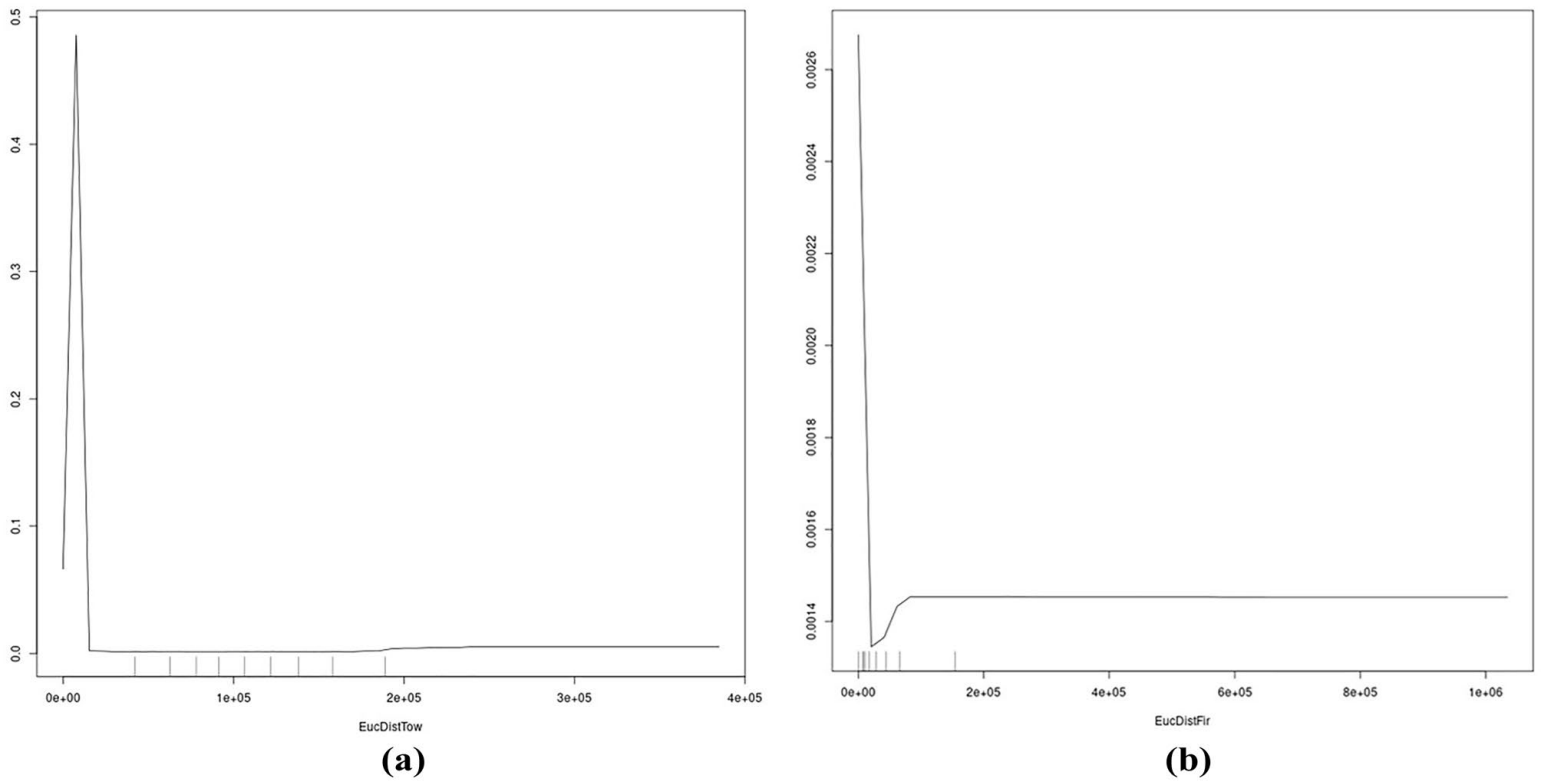
(**a**,**b**) Partial dependence plots of top two predictors using node purity (EucDistFir, EucDistPipe; the other two partial dependence plots of this group are already shown in Fig. 6).

For a wider inference, it becomes clear from Fig. 4 that a multivariate set of ecological predictors—at least 20—drives the occurrence of GGOWs in Alaska, not just a few single predictors but a wider range of predictors together across a wide environmental spectrum interacting in synergy. Whereas, a parsimonious approach does not capture GGOW’s distribution in Alaska and must be biased adding variance. However, seen from that angle, the predictor group that is directly related to human impacts and urbanization stands out (Figs. 4 and 5), whereas the more typical ecological niche predictors like climate and landcover seem to play a much lower role and are overruled by human/urban predictors. Figures 4, 6 and 7 make clear that GGOWs are found in habitats with a high human footprint, and/or occur next to it, but usually not far away from them or in the remote wilderness. Lakes and fires (^54^ for underlying ecology see^55-57^) could be a secondary, weak relationship for GGOW habitats. The predictors of Distance to coast and Proximity to Airports deserve more attention (many predictions are in coastal areas, a few GGOW presence records come from the Federal Bird Strike airport database (https://wildlife.faa.gov/); as per^43^). The predictors related to human cities and towns, human footprint, distance to pipeline and human density are among the leading predictors for GGOWs, out of a diverse set of 100 predictors overall (their variable importance ranks are shown in Fig. 4). GGOWs are known to rely on small mammals for prey (e.g.^58^). But noteworthy in our model findings is the high rank of the predictor called ‘model 1’, which is the predicted range of the 60+ bark beetle species community^59^. The correlation of GGOWs with bark beetles is a new finding, have never been described before (see^60^ for a traditionally reported small mammal link) and should be pursued more in future research projects.

What is the meaning of ‘background’ in binary presence/pseudo-absence models? Here we model binary predictions in the absence of ‘confirmed absence’ data points for this species (as shown in^47,60^). However, while meaningful absence data is missing for GGOWs in Alaska, e.g. a Breeding Bird Atlas, here we use a 1 km sample from all of Alaska and its diverse habitats making it a next-to-perfect comparison with the best-available presence records of GGOWs^61^, covering a unique time period 1880–2019.

We explain the mismatches with traditional GGOW maps due to lack of data, some parsimony perspectives and methods, previously insufficient predictor sets realized, and plain human expert assessment and perception errors^11,62^. The ML/AI methods we present as a Super SDM can help to overcome those problems. It also disproves the ‘human-desired’ distribution range of the ‘Phantom of the North’. At minimum, it shows a quantified and testable predicted ecological niche for GGOW to work from, and such a repeatable workflow.

How good and valid are the predictions achieved?

**Figure 8:**
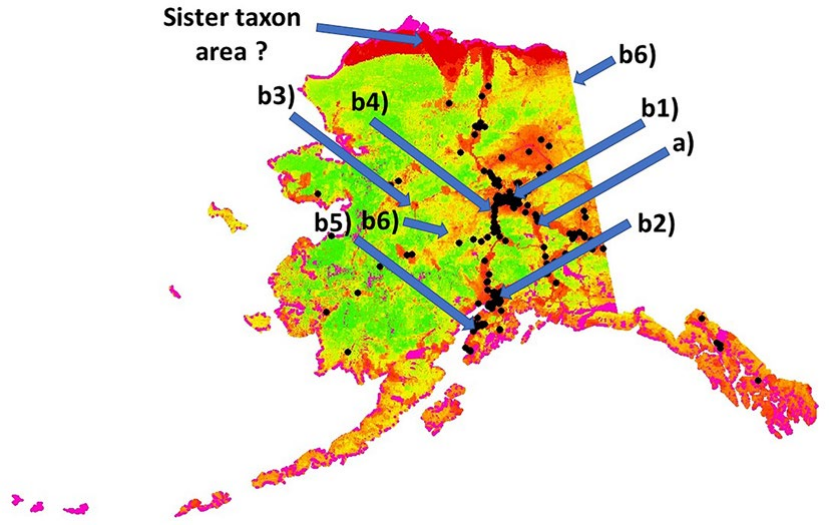
GGOW predictions from the RF model run in ‘the cloud’ supercomputing overlaid with the training data (black dots). In addition, alternative Great Gray Owl sightings are overlaid (**a**) Detailed field assessment from Andrews (2019), and (**b**) recent sightings of the last 4 years from citizen efforts like birding listservers (b1,b2), and iNaturalist (b3–5) and Xeno-Canto (b6; 2 entries). It represents app. an additional 1% of the training data available for this ‘elusive’ species.

Using the Receiver Operating Characteristic^11,64,65^, our internal prediction accuracy shows a ROC value of over 90% for Alaska’s lattice points, but as provided by the software as a standard performance metric^11, and citations within^). Alternative assessment data are more powerful but few (see overview in^43^ for GGOW). However, as shown in Fig. 8, the existing ones at least fully confirm the model for the survey areas with high accuracy; the model predictions match the training data ‘very well’ (= almost a 100% match for locations tested) using recent bird watching records and iNaturalist records, extending the data set of c. 1% of the training data.

GGOWs are widely described as species for ‘the taiga’, e.g. in Google. Thus far, there are not many GGOW records for Alaska beyond the Brooks Range and the Arctic Tundra but some exist (Fig. 5 and evaluation data; Fig. 8). However, already in adjacent Canada, and in the Old World GGOWs are reported at those latitudes and at higher Northern latitudes. A sound recording was made in the Arctic area that we predict (for Alaska-Canada-border see https://xeno-canto.org/species/Strix-nebulosa). While prey abundance is generically high in those areas, thus far it is not known whether the model output predicts there the realized niche or indicates a sister taxon, e.g. snowy owl? Arguably, with an increased shrubification of the Arctic the boreal ecosystem is already moving north allowing for perch sites of GGOW with prey

Overall, the prediction results from the workflow we present—thus far—are difficult to beat for evidence, or to show wrong with empirical data at hand (see Fig. 8 below). They are far from overprediction, e.g. for wilderness and protected areas. Until there is better data available, specifically GGOW presences and absences, or nest, migration and telemetry data and expert information for GGOW are provided open access (e.g. from NGOs or governmental records), our results remain as good as they get and are to be used for management for time to come. All data are publicly available for that reason and allow for extension,  assessments, updates and improvements as needed in a quantified open access fashion.

## Discussion

Here we present for the first time the best-available Open Access data for the Great Gray Owl (GGOW) as well as its 100+ geographic information system (GIS) habitat predictors for Alaska with ISO compliant metadata for a public audience. This presents the largest and most modern data set (“Big Data”) ever compiled for this species, its environment, and the state of Alaska (= the area in the U.S. with the largest wilderness and protected area system left) covering data from 1880 to 2019 and beyond (assessment data 2019 onwards).

Further, we were able to run the first Alaska-wide Super SDM model of GGOW predictions from such data. Super SDMs can have limitations dependent on data used, should always be assessed with several lines of independent evidence. They are not the ultimate and final statement on species-habitat associations, but they come close^34^. At minimum, they are low-cost rapid assessments capturing data quantitatively in time and space. It also is a great leap forward to be more ecological and more inclusive of all information and synergies available setting a new stage for species-habitat assessments^11^.

Beyond the data provided, the other strength of this work consists of the conceptual use and workflow of an ensemble model applied in a powerful cloud computing (supercomputer) environment, allowing for overcoming a traditional computational bottleneck using 100 predictors for new findings that were not able to be achieved before for inference. Overcoming the technical limitations of memory that come with the traditional computing environment allowed here a showcase for new computational and biological insights and progress, e.g. that GGOWs associate consistently with a high human footprint.

We followed the approach by Leo Breiman^48,49^ to infer from the prediction, as well as Jerome Friedman (cited in^11,30^) ‘*many weak learners create a strong learner*’. The actual base-code was made available (see Data Availability section, Appendix 4 within) for improvements, and the results were mapped in Open Source GIS for further use and application. Arguably, these ML models can be tested, improved and extended in various ways (for instance, the randomForest in R version can usually be challenged by Leo Breiman’s code in the Minitab Salford Predictive Modeler System (https://www.minitab.com/en-us/products/spm/). But here we show a proof of concept with all settings allowing to run and establish Super SDMs in a quantified and testable fashion.

We further pursed the concept of data mining, which keeps raw data and potential outliers ‘as is’, because that is a more powerful approach to the vast and otherwise accurate dataset. It leaves the actual ML algorithm to resolve problems and find the best prediction, rather than a biased human perception, assumptions, human errors^11,65,66^, and human meddling with a wrath of data and model settings within a complex ecological setting widely not understood (^23,63,68^; see^11,65^ for alternatives). The same applies to the concept of overfitting (better to be referred to as a full fit, as per^11^); randomForest is designed on the principle of ‘bagging’ which tends to avoid overfitting in the default setting, including a robust handling of outliers and autocorrelation^11^.

Biologically, it is known that GGOW’s populations and subsequent habitat needs are somewhat cyclic^58,66–68^; here we present the year-wide average ecological niche across decades of observations with a testable and quantified prediction. From the raw data and predictions one can already easily show that GGOW is not a ‘*phantom of the north*’ (^38^, see also^69^) but instead it is a circumpolar species occurring instead in more southern areas^70,71^, e.g. in coastal areas and latitudes of 40 degrees North^72-77^ and thus living already for a long time in a highly urbanized, industrial, forestry and farming landscape among humans in the “Total Anthropocene” (^78^; for specific GGOW examples in its range see^79-86^). GGOWs do associate with a high human footprint. In Alaska, albeit well known and enthusiastically reported^87-89^, the GGOW is quite a rare sighting as such, but it is clearly affiliated with human landscapes^43^. However, a solid description and effective GGOW conservation plan with an associated budget for this species exist elsewhere (see^90^ for Oregon,^91,92^ for national forest practices) but is widely missing in (urban) Alaska (^93,94^; see^95-100^ for specific GGOW field protocols to be used; see^101^ for Alaska). Using a Super SDM, here we further can infer^102^ and confirm that GGOW in Alaska (= the state with the biggest wilderness in the U.S. and holding its largest national park system) is in essence an urbanized bird that associates with industrial infrastructure, pipeline, roads, urbanized centers and farming. Whereas the vast tracts of Alaska, e.g. western Alaska, interior Alaska and protected areas are widely free of reported GGOW sightings and high numbers/clusters (that is true for raw data as well as for the predictions of the ecological niche using over 100 predictors). Essentially, our finding flips how this species must be perceived and managed (e.g. opposite from^81,103^). As a minimum estimate, we find GGOW is an urbanized species primarily detected thus far in association with humans and man-made habitats (^104^; this habitat link can somewhat cycle over the years, and it is even stronger during migration and in wintering areas, such as found for a long time already in Alberta and Manitoba/Canada;^72,95, 105, 106^, and in the Old World^107^; contrast it with^93^). A question remains for GGOWs in the high arctic, and whether it occurs there much, or is a sister taxon like the Snowy Owl occupying that niche? Arguably, prey is abundant for GGOW and so are perching options.

How generalizable are the ecological niche predictions for inference, and for the realized niche? In the wide absence of any relevant research design specific for GGOW (see^108-110^ for road bias and how resolved), representative sampling, of an Alaskan Bird Atlas and Nesting Survey for that matter (compare with Birds of Yukon^111^, or bird banding/ringing work elsewhere in the GGOW range, e.g.^112^), and unsubstantiated narratives^113^ this question currently cannot be answered with ultimate accuracy (compare with^114^; see^101^ for owls in Southeast Alaska). Table 3 shows that more data and information exist that actually could be used, but unfortunately it is not presented to us, communicated with the public, and available to the public or science’s use. However, it is clear that much avian and raptor research was done but not shared, and thus opportunity was left unused, which is a generic pattern in wildlife-related research, specifically in Alaska, and for ML/AI applications (see for instance^11,115, 116^). As SDMs can indeed generalize^11,28^ here we used all publicly available GGOW information human-possible to-date in order to achieve the goals starting from 1880 onwards.

**Table 3:** Data sources for Great Gray Owls in Alaska.

Data source name	Content^a^	Open access	Used in study	Notes
GBIF	Presence	Yes	Yes	Training Data
Alaska Museum	Presence	Partly	No	Partly in GBIF already, incomplete data set of specimen only
eBird	Presence	Yes	Yes	Training Data
Birdwatch List-server	Presence	Yes	Yes	Training and Assessment Data
iNaturalist	Presence	Yes	Yes	Assessment Data
Bird Banding	Presence	No	No	Not easily available, few locations, e.g. EURING-BTO, USFWS, CWS Bird Banding Atlas
Xeno Canto	Sound/Presence	Yes	No	Recording exist for Alaska-Canada Arctic boundary area, as well as near a village
Feederwatch	Presence	Partly	No	Insufficient coverage for Alaska
Xmas Bird count	Presence	Partly	No	Limited value for spatial coverage
Movebank	Presence	No	No	Not shared, no coverage for Alaska
State & Federal Agencies	Presence	No	No	Not shared, not findable, some coverage for Alaska
Commercial Experts/contractors and NGOs	Presence/Abundance	No	No	Unknown amount of research, data and expertise
Raptor Biologists/Falconers	Presence/Abundance	No	No	Entire Professional Raptor and Wildlife Societies do not share or truly promote Open Access data sharing for many years^b^

^a^'Presence' refers to an implied georeferenced location; absence is not considered, yet. Often data include other information like abundance or attributes but which are not used here. The use of telemetry, data logger, nest and survey data are essential for such records.

^b^Many of such data works and funding are often coming from public environmental impact studies and contracts, e.g. for wind farms, mining and oil & gas projects, and airport strike risk assessments working on, and with, public resources.

While our model prediction assessments are ‘high’, arguably our model prediction still presents an underestimate of reality and an incomplete truth; many pixels await ground-truthing. Already the limits of data, research design and pseudo-absences can potentially limit inference (e.g.^117^). Cycling aspects of the Arctic and its populations are not included yet (e.g.^118,119^) and more focused data will fill other gaps and provide model updates. However, it is undeniable—from the raw data and the predictions alike—that GGOWs occur in human-dominated areas of Alaska. Those sightings are linked with man-made, urban and industrial habitats indeed, beyond ‘myth’. It matches other wildlife research findings in Alaska, such as^50^.

This research sets the stage for how habitat models—SDMs—can be run and improved. Leaving out predictors in the pursuit of parsimony  is still widely done in most of the species-habitat works in Alaska to-date—must be seen as willful, with an untested hypothesis-drop, that knowingly creates uncertainty and bias, leaving out many possible questions unanswered (see^11,117, 118^ for a vast range of applications). In the light of Super SDMs, such scholastic work must be perceived as ignoring best-available options; arguably it has either not done its homework or does not want to use existing data, information and employ easily available potential at hand for their research while better approaches have existed for many decades (see^57,120–124^ for other applications done in Alaska, and see^125-131^ for other disciplines).

As commonly done in wildlife applications, e.g.^11,132^, here we show a ‘proof of concept’ with first inference. It is primarily technical progress it allows for bigger impacts on improved inference related to species and habitat management, in Alaska and globally. Here we were able to set a new available and mandatory baseline for inference: we established the Super SDM. Having such concepts available allows for predictions of high accuracy (see^132^ for 1 m prediction resolution), specifically when it comes to impact assessments, e.g. with an optimized survey design^133^, done into the future and with climate change (e.g.^134-136^). For Alaska, coming already from a troubling industrial past (e.g.^137^), much more industrial development is the current path to come in the Anthropocene. It is where state-wide mining and nuclear reactors are now tried and planned while the permafrost landscape melts, and the boreal forest gets cut down and burns^55,138^, with a new major sector exponentially on the rise—seabed mining^139^. As the decaying fate of natural resources and wilderness has shown^140,141^, regular ‘modern’ conservation governance has widely failed in Alaska and beyond (^12^; see for instance Alaska’s salmon crisis including King Salmon disappearance within just less than 50 years under such a regime affecting habitats and associated thousand-year long indigenous cultures relying on it^142,143^). Here we provide some quantified progress on best-available human options for global sustainability.

## Data Availability

Data are shared Open Access, as per Methods and Appendix at the following URL https://drive.google.com/drive/u/0/folders/1rz3ZW3xplvdEf8LDu-d7-1BDXF6XxNMY, and also available from the authors on request.
